# The Individual Progress Test of Gynecology and Obstetrics Residents (TPI-GO): The Brazilian Experience by FEBRASGO

**DOI:** 10.1055/s-0041-1731803

**Published:** 2021-07-27

**Authors:** Marcos Felipe Silva de Sá, Gustavo Salata Romão, César Eduardo Fernandes, Agnaldo Lopes da Silva Filho

**Affiliations:** 1 Faculdade de Medicina de Ribeirão Preto, Universidade de São Paulo, Ribeirão Preto, SP, Brazil; 2 Universidade de Ribeirão Preto, Ribeirão Preto, SP, Brazil; 3 Faculdade de Medicina do ABC, Santo André, SP, Brazil; 4 Faculdade de Medicina, Universidade Federal de Minas Gerais, Belo Horizonte, MG, Brazil

The great expansion of Medical Residency Programs in Brazil in recent decades has made it difficult to properly evaluate the trained professionals and the quality of training offered.

Today, ∼53,776 physicians are registered at the National Medical Residency Commission enrolled in 4,862 Medical Residency Programs offered by 809 institutions. Only in Gynecology and Obstetrics (Ob-gyn), there are 312 Medical Residency Programs. Despite the recommendation that resident physicians undergo quarterly theoretical and practical evaluations by the programs, this has not been happening regularly. Therefore, there is no information about the performance of residents during their training and the quality of training programs. Evidently, this knowledge should be based on information obtained during ongoing evaluations of the programs, with visits, audits and reports, although this has not happened in practice.

According to current legislation, specialists graduated from Medical Residency Programs approved by the National Medical Residency Commission automatically receive the specialist certificate recognized by the Ministry of Education and the Federal Council of Medicine without any evaluation process of the students' competences at the end of the program.

In view of the fragility of the system, the Medical Specialties Societies in Brazil, with special authorization from the Brazilian Medical Association, started to grant the title of specialist for graduates of residency programs after an evaluation through a flexible exam, depending on the Society granting it. In this sense, the Title of Specialist in Gynecology and Obstetrics (Portuguese acronym: TEGO) given by the Brazilian Federation of Gynecology and Obstetrics Associations (FEBRASGO) is only granted upon evaluation of the candidate through theoretical and practical tests carefully prepared by the National TEGO Commission of FEBRASGO.

In this evaluation process, important failures in the training of new specialists who completed the residency program have been observed, which has raised the failure rates for the TEGO. Thus, considering the need to qualify the training of Ob-gyn specialists in Brazil and understanding that the Individual Progress Test (IPT) represents a great reference for the self-assessment and improvement of resident physicians and Medical Residency Programs, as of 2018, FEBRASGO has implemented the Individual Progress Test for Ob-Gyn Residents (IPT-GO). Although the IPT is widely used internationally, in Brazil there is little experience reported on its use in Medical Residency.

The IPT is a comprehensive assessment that preferably uses multiple choice questions and is periodically applied to all students of the same curriculum or program, 1 aligned with modern constructivist education and promotes long-term knowledge. The longitudinality of this modality of assessment provides a unique and demonstrable measure of students cognitive progression. 2

The functional purpose of the IPT is to provide reliable information for self-assessment of candidates and service providers of Medical Residency Programs hence, it is a formative assessment. For resident physicians who take the test, the IPT provides an accurate measure of their level of knowledge in relation to their peers and in relation to the final objectives of the specialty training, according to the Gynecology and Obstetrics Competence Matrix. 2 3

Furthermore, through the performance in serial evaluations, the individual progress of the cognitive component can be evaluated. After each assessment, it is possible to reaffirm and consolidate knowledge and identify learning gaps and points to be improved. 4

For preceptors and supervisors of Medical Residency Programs, the IPT-GO provides information on the performance and progression of residents who have taken the exam. Through this information, the profile of residents admitted to each service, the added knowledge of residents throughout the training program and the level of knowledge of graduates in relation to the national average and in relation to the objectives of the Competence Matrix can be assessed. It also allows the identification of strengths and points of improvement or areas requiring reinforcement for learning. 4

Since 2018, the IPT began to be offered annually to all resident physicians in the first (R1), second (R2) and third years (R3) of training regularly enrolled in Medical Residency Programs recognized by the Ministry of Education.

In 2018 and 2019, the test was applied in person and simultaneously in 11 Brazilian cities in regions with the highest concentration of Ob-Gyn Residency Programs. In 2020, due to the Covid-19 pandemic and in compliance with sanitary requirements, the IPT could not be applied in person. Faced with this new challenge, the choice was to apply the test online, since this measure is supported by the literature. 5

The theoretical test for obtaining the TEGO, applied annually to newly graduated specialists in the field, is a comprehensive assessment of the skills provided for in the Gynecology and Obstetrics Competence Matrix 3 and used as a reference to the final level of Residency Programs. For these reasons, this test model was chosen to be applied in the IPT-GO. 6

Criteria ranging from a bonus of points to exemption from the TEGO theoretical test in the year following the completion of the residency program were established as a form of encouraging residents' participation in the IPT-GO. These criteria are based on adherence and individual performance on the test. To be entitled to bonuses, it is an essential condition that the resident participates in all three versions of the IPT as R1, R2 and R3, with a progressive minimum performance established in accordance with the competition notice published each year. 7

## Individual Feedback to Candidates

Feedback on the results of each candidate's performance in the IPT-GO is provided confidentially through a password-protected online system with personal access. The aim of this measure is to avoid embarrassment, discrimination or disqualification of candidates with unsatisfactory performance. Therefore, this evaluation is not intended to rank candidates or services. The online system presents graphs where candidates can assess the progression of their individual performance and compare it to their peers through the median (Me), 30 ^th^ percentile (P30) and 60 ^th^ percentile (P60) of the overall performance. For candidates completing residency programs (R3), in addition to the evaluation of the performance level in the triennium, information on whether or not they have received a bonus for TEGO of the following year is also provided.

## Feedback to Services and Residency Programs

For Medical Residency Programs, feedback is provided by FEBRASGO directly to preceptors or responsible persons. The information provided corresponds to the median performance of each category of residents (R1, R2 and R3) of the service compared with the Median, P30 and P60 values of the overall performance without identifying the residents.

## Analysis of Results

In 2020, the IPT-GO completed its third edition, making it possible to assess the performance of the first complete cohort of residents who took the test in the three consecutive years of Ob-Gyn Residency Programs in Brazil ( Table 1 ).

**Table 1:** Number of participants in the three Residency levels over the three years of application of the Individual Progress Test

Level	2018 n (%)	2019 n (%)	2020 n (%)
R1	497(41)	568(43)	628(44)
R2	360(30)	457(35)	480(34)
R3	345(29)	289(22)	314(22)
Total	1202	1314	1422
Increment		9.3	7.6

Among candidates, female participation was 85.6%, 85.9% and 85.5% respectively in 2018, 2019 and 2020, in a clear demonstration that Ob-Gyn have become a predominantly women's specialty. The total number has increased over the three years, particularly due to R1 increment, since a decrease in R2 and R3 was observed. Some factors should be mentioned in this “balance” of gains and losses in these numbers. Undoubtedly, the bonus and exemption opportunities in the TEGO theoretical test have contributed to encourage longitudinal participation of residents. However, a large part of the discontinuity may be attributed to the poor performance of some candidates in the first versions of the test, resulting in dropouts due to the impossibility of bonus or exemption from the TEGO theoretical test. Another noteworthy factor that justifies non-adherence a considerable part of the residents would be the difficulty of program coordinators in releasing candidates on the day of the exam, considering the resident's need for work given the composition of the teams on duty at the respective hospital. As demonstrated, in all editions of the IPT-GO there was a progressive improvement in performance among candidates from the first (R1), second (R2) and third (R3) years of the Ob-Gyn Residency Program. This reflects the knowledge increase occurring throughout the training period in most programs. It also reflects the quality of the test, which consists of a comprehensive, valid and reliable assessment of all knowledge expected by those completing the program (R3) ( Table 2 ).

**Table 2:** Median (Me), 30 ^th^ (P30) and 60 ^th^ (P60) percentiles of the grades assigned to residents at different levels who took the Individual Progress Test-GO in 2018, 2019 and 2020

	Progress Test - Performance of Gynecology and Obstetrics residents by level of treatment								
	2018			2019			2020		
	R1	R2	R3	R1	R2	R3	R1	R2	R3
P30	4.8	5.2	5.5	5.5	5.8	6.1	5.1	5.4	5.8
Me	5.2	5.6	5.9	5.9	6.2	6.6	5.5	5.8	6.3
P60	5.5	5.8	6.1	6.1	6.4	6.8	5.6	6.0	6.5

## Performance of the Cohort of Residents from 2018 to 2020

The performance evaluation of 314 resident physicians who started the IPT-GO in 2018 as R1 and completed the last version in 2020 deserves attention. This cohort of candidates represents the first group that underwent the three versions of the IPT-GO since its implementation, and serves as a reference for various analyzes and considerations.

The median grades of performance of this group in 2018, 2019 and 2020 were respectively 5.2(R1) 6.2(R2) and 6.3(R3). A progression in the performance of the same group of residents from the beginning to the end of their training period can be observed, reflecting the acquisition of knowledge during the residency program. These results are consistent with a well-structured IPT, which is comprehensive and focused on the content expected for graduates of the Program. 2

The smaller increase in performance between years 2019 and 2020 may somehow be a reflection of the Covid-19 pandemic period that certainly affected the performance of all residents indistinctly, given the huge restrictions imposed on in-person activities, particularly in outpatient and surgical practices. 8

In conclusion, the implementation of the IPT for Ob-Gyn residents across the country was a great experience in several aspects.

The wide dissemination of the IPT on social networks, on FEBRASGO's institutional Web site, at scientific events in the specialty and through direct mail to all Ob-Gyn residents and Medical Residency Programs in the country allowed FEBRASGO to get closer to residents, improve and update the registration of Medical Residency Programs, preceptors and other relevant information. The number of residents associated with FEBRASGO has increased considerably in the last two years.

The form of performance feedback to program coordinators allowed for the comparison of results with similar services throughout the country and motivated reflections and internal debates among coordinators, preceptors and residents, and drew attention to the need for improvement in many programs. Note that institutional commitment through the support of program directors, coordinators and preceptors is key, as there is a need to release residents from the shift schedule on the day of the test.

Information from the Individual Progress Test can be used consistently for diagnostic, prognostic and corrective learning through self-assessment and structured feedback. When compared with final summative evaluations, the IPT provides greater support and security in making high-impact decisions such as approval, failure and progression of the student. Additionally, longitudinal data can serve as a measure of the quality and transparency of programs and achievement of curriculum objectives by educational institutions.

Considering the immense difficulties encountered by government agencies to audit Medical Residency Programs on a permanent basis, this model may be adopted and validated by the National Medical Residency Commission as one of the parameters for evaluating Medical Residency Programs through partnerships with the Medical Specialties Societies. It is worth mentioning that all expenses arising from the application of the test were made with FEBRASGO's own resources so to not burden the National Medical Residency Commission.
